# Cipatrijugin G, a new trijugin-type limonoid bearing an uncommon *γ*-hydroxybutenolide unit from the aerial parts of *Cipadessa cinerascens*

**DOI:** 10.1007/s13659-013-0074-z

**Published:** 2013-11-06

**Authors:** Cheng-Shi Jiang, Yan Li, Zhen-Zhong Wang, Xiao-Yin Huang, Wei Xiao, Yue-Wei Guo

**Affiliations:** 1 State Key Laboratory of Drug Research, Shanghai Institute of Materia Medica, Chinese Academy of Sciences, Shanghai, 201203 China; 2 Jiangsu Kanion Pharmaceutical Co. Ltd., Lianyungang, 222001 China

**Keywords:** *Cipadessa cinerascens*, trijugin-type, limonoid

## Abstract

A new trijugin-type limonoid, cipatrijugin G (**1**), together with the related known cipatrijugin A (**2**), were isolated from the aerial parts of *Cipadessa cinerascens*. The structure of the new compound was determined by extensive analysis of its spectroscopic data and by comparison of its NMR data with those reported in the literature. Compound **1** showed cytotoxicity against tumor cell line A549 with an IC_50_ value of 9.78 μM. This is the first report of a trijugin-type limonoid bearing a *γ*-hydroxybutenolide unit, in comparison to the common furan unit as ring E.

**Electronic Supplementary Material:**

Supplementary material is available for this article at 10.1007/s13659-013-0074-z and is accessible for authorized users.

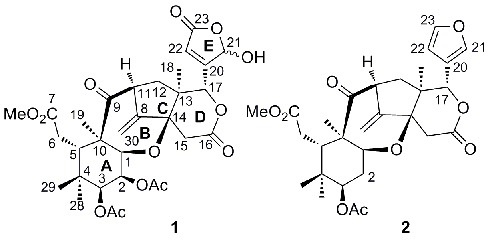

## Introduction

Plants belonging to family Meliaceae are an abundant source of limonoids, which are a structurally diverse group of natural products, with a highly complex polycyclic skeleton.[1–4] These unusual structural features have attracted great attention as a challenging target for total synthesis,[5,6] bioactivity evaluation,[7,8] and biosynthetic studies.[9,10] The genus *Cipadessa* (Meliaceae) is composed of nine species of shrubs or small tress (up to 5 m tall). *C. cinerascens* (Pell.) Hand.-Mazz. is a medicinal plant widely distributed in the southwest of mainland China. The leaves and roots of the plant have traditionally been used for the treatment of rheumatism, malaria, scalds, and skin itches.[11] Previous phytochemical investigations of *C. cinerascens* led to the isolation of a series of limonoids, some of which showed various biological activities, including cytotoxicity, insecticidal, and trypanocidal activities.[1,12]

In the course of our ongoing project searching for bioactive secondary metabolites from Chinese medicinal plants,[13–17] with *Cipadessa cinerascens* was recently collected from Guangxi Zhuang Automomous Region, China. A chemical investigation of the aerial parts of the plant has resulted in the isolation of a new trijugin-type limonoid, named cipatrijugin G (**1**), together with a related known compound cipatrijugin A (**2**)[18] (Figure 1). In this paper, we report the isolation and structure elucidation of the new compound **1**.

*To whom correspondence should be addressed. E-mail: wzhzh-nj@163.net (W. Xiao); ywguo@mail.shcnc.ac.cn (Y.W. Guo)

**Figure 1.** Chemical structures of compounds **1** and **2**

## Results and Discussion

The air-dried powdered aerial parts (2.5 kg) of *C. cinerascens* were extracted using MeOH at room temperature. The MeOH extract was partitioned between a $CHCl_3$ and $H_2O$ phase. The $CHCl_3$-soluble phase was separated using silica gel column to obtain twelve fractions, named A–L. Fraction I was repeatedly chromatographed over silica gel, and Sephadex LH-20 columns to isolate compounds **1** and **2**.

The known compound **2** was readily identified as cipatrijugin A by comparison of its spectroscopic data with those reported in the literature.[18] Moreover, cipatrijugin G (**1**) was obtained as a white powder. The molecular formula, $C_{31}H_{38}O_{13}$, was determined using HRESIMS from the quasi-molecular ion peak at $m/z$ 641.2205 $[M + Na]^+$ (calcd 641.2210), suggesting the presence of thirteen degrees of un-saturation. The IR spectrum showed the absorptions indicative of hydroxyl (3442 $cm^{-1}$) and ester (1742 $cm^{-1}$) and unsaturated carbonyl (1686 $cm^{-1}$) functionalities. The $^1H$ and $^{13}C$ NMR

**Table 1.** $^1$**H and** $^{13}$**C NMR data for compounds 1**$^a$ **and 2**$^b$ **(*J* in Hz)**

| position | 1 | | 2 | |
|---|---|---|---|---|
| | $\delta_H$ | $\delta_C$ | $\delta_H$ | $\delta_C$ |
| 1 | 4.19, d, (3.3) | 73.7 (d) | 4.16, t, (2.5) | 71.6 |
| 2α | 5.22, t, (3.3) | 65.3 (d) | 2.19, dt, (16.0, 2.5) | 29.6 |
| 2β | | | 1.91, dt, (16.0, 2.5) | |
| 3 | 5.15, d, (3.6) | 74.1 (d) | 4.79, t, (2.5) | 74.7 |
| 4 | | 39.3 (s) | | 38.3 |
| 5 | 2.90, overlapped | 37.8 (d) | 2.98, t, (4.5) | 37.9 |
| 6α | 2.95, br. d, (4.2) | 29.2 (t) | 2.90, dd, (18.0, 4.5) | 29.6 |
| 6β | 2.25, br. d, (3.6) | | 2.27, dd, (18.0, 4.5) | |
| 7 | | 176.2 (s) | | 174.4 |
| 8 | | 142.7 (s) | | 144.5 |
| 9 | | 208.5 (s) | | 210.2 |
| 10 | | 56.0 (s) | | 55.2 |
| 11 | 3.60, dd, (3.3, 9.3) | 58.3 (d) | 3.52, dd, (4.0, 10.0) | 58.6 |
| 12α | 1.85, m | 35.3 (t) | 1.70, dd, (14.0, 10.0) | 35.7 |
| 12β | 2.80, overlapped | | 2.81, dd, (14.0, 4.0) | |
| 13 | | 45.5 (s) | | 46.0 |
| 14 | | 87.6 (s) | | 87.5 |
| 15α | 2.88, overlapped | 33.7 (t) | 2.79, m | 34.5 |
| 15β | 2.80, overlapped | | 2.79, m | |
| 16 | | 167.5 (s) | | 170.7 |
| 17 | 6.48, s | 78.4 (d) | 6.41, s | 79.3 |
| 18 | 0.91, s | 17.7 (q) | 0.78, s | 17.5 |
| 19 | 1.08, s | 19.1 (q) | 1.02, s | 19.9 |
| 20 | | 164.1 (s) | | 121.9 |
| 21 | 6.18, br. s | 96.1 (d) | 7.55, s | 143.5 |
| 22 | 6.31, s | 119.9 (d) | 6.46, br. s | 108.6 |
| 23 | | 169.2 (s) | 7.44, br. s | 139.8 |
| 28 | 0.78, s | 27.2 (q) | 0.87, s | 27.5 |
| 29 | 1.05, s | 22.6 (q) | 0.95, s | 22.9 |
| 30α | 5.50, s | 115.8 (t) | 5.37, br. s | 114.1 |
| 30β | 5.25, s | | 5.17, br. s | |
| Ac-2 | 2.05, s | 20.7 (q) | | |
| | | 170.3 (s) | | |
| Ac-3 | 2.05, s | 20.2 (q) | 2.04, s | 20.8 |
| | | 170.9 (s) | | 168.8 |
| OCH3 | 3.68, s | 52.6 (q) | 3.66, s | 51.7 |

$^a$ $^1$H NMR ($CDCl_3$, 400 MHz); $^{13}$C NMR ($CDCl_3$, 100 MHz); Chemical Shits (ppm) referred to $CDCl_3$ ($\delta_H$ 7.26, $\delta_C$ 77.0,).
$^b$ $^1$H and $^{13}$C NMR data of **2** from ref. 18.

spectra of **1** (Table 1) exhibited resonances for two acetyl groups ($\delta_H$ 2.05, 6H; $\delta_C$ 20.7, 20.2, 170.3, 170.9), four quaternary methyl ($\delta_H$ 0.91, 1.08, 0.78, 1.05; $\delta_C$ 17.7, 19.1, 27.2, 22.6), one carbomethoxy ($\delta_H$ 3.68; $\delta_C$ 52.6, 176.2), one *exo*-cyclic double bond ($\delta_H$ 5.50, 5.25; $\delta_C$ 115.8, 142.7), one ketonic carbonyl ($\delta_C$ 208.5), one ring-D lactone, one hemiactal group ($\delta_H$ 6.18, $\delta_C$ 96.1), and four further oxygen-bearing methines ($\delta_H$ 4.19, 5.15, 5.22, 6.48; $\delta_C$ 65.3, 73.7, 74.1, 78.4). The aforementioned spectroscopic features and comparison with co-occurring cipatrijugin A (**2**)[18] strongly suggested that **1** was a limonoid with trijugin-type skeleton. In fact, the $^1$H and $^{13}$C NMR data of **1** are very similar to those of compound **2** in rings B, C, and D, and mainly differed from those of **2** for C-20, C-21, C-22 and C-23 in ring E by the presence of a γ-hydroxybutenolide unit (instead of a furan unit), as well as an additional acetoxyl group at C-2 in ring A. Although there is no detected HMBC correlation from H-2 to the acetyl carbonyl carbon in OAc-2, the location of acetoxyl group at C-2 in ring A can be deduced by the remarkable downfield shifts of H-2 ($\delta$ 5.22) and C-2 ($\delta$ 65.3), and $^1$H-$^1$H COSY correlations of H-2 (5.22, t, $J$ = 3.3 Hz)/H-1 (4.19, d, $J$ = 3.3 Hz), and H-2 (5.22, t, $J$ = 3.3 Hz)/H-3 (5.15, d, $J$ = 3.6 Hz). The γ-hydroxybutenolide unit for ring E could be confirmed by diagnostic HMBC correlations of H-21/C-22/C-23, and H-22/C-20/C-23. Furthermore, the 21-hydroxy-20(22)-ene-21,23-γ-lactone moiety was unambiguously secured by comparison of the $^{13}$C NMR data with those of the corresponding part of the model compound turrapubesin G, a natural product previously isolated from *Turraea pubescens*.[19] The HMBC correlations of H-22/C-17 and H-17/C-20 clearly indicated a linkage between rings D and E. Finally, comprehensive analysis of the $^1$H-$^1$H COSY, HSQC, and HMBC spectra (Figure 2) allowed to assign all the chemical shifts in the $^1$H and $^{13}$C NMR spectra (Table 1) of **1**.

**Figure 2.** Selected $^1$H-$^1$H COSY (▬), HMBC (⟶), and ROESY (↶ ↷) correlations for **1**

The relative configurations for ring junction centers of **1** were suggested to be the same as those of co-occurring **2** based on similar $^1$H and $^{13}$C NMR chemical shifts and biogenetic consideration. Further, the relative configurations for C-2 and C-17 were determined by ROESY experiment.

Thus, the relative configuration of C-2 was established by the clear NOE cross peak of H-2 ($\delta_H$ 5.22)/Me-19 ($\delta_H$ 1.08), suggesting the $\beta$-orientation for acetoxyl group at C-2. The $\beta$-orientation of H-17 was indicated by the NOE correlation of $H_\beta$-12 ($\delta_H$ 2.80)/H-17 ($\delta_H$ 6.48), consequently establishing the $\alpha$-orientation for $\gamma$-hydroxybutenolide ring at C-17. Thus, the relative structure of **1** was demonstrated as depicted in Figure 2.

Compounds **1** and **2** were tested for *in vitro* cytotoxicity against tumor cell lines A549 and HL60. The results indicated that compound **1** showed strong cytotoxicity against A549 with an $IC_{50}$ value of 9.78 μM and no inhibition on HL60.

Trijugin-type limonoids are of particular interest due to their complicated structures and promising activities. Since their first isolation in 1987,[20] about 21 analogues had been reported to date according to the literature survey. However, to the best of our knowledge, cipatrijugin G (**1**) presents the first example of trijugin-type limonoids bearing a $\gamma$-hydroxybutenolide unit, instead of the common furan unit as ring E. The discovery of **1** is an important addition to a diverse and complex array in the rapidly expanding class of known trijugin-type limonoids. Further studies should be conducted to prove the biosynthetic origin of these compounds and to understand their real ecological roles played in the life cycle of the plant, as well as to confirm their absolute configuration by total synthesis.

## Experimental Section

**General Experimental Procedures.** Optical rotations were measured on a Perkin-Elmer 341 polarimeter. IR spectrum was recorded on a Nicolet Magna FT-IR 750 spectrometer. NMR spectra were measured on a Varian Mercury-400 spectrometer with the residual $CDCl_3$ ($\delta_H$ 7.26 ppm; $\delta_C$ 77.01 ppm) as an internal standard. EIMS and HREIMS spectra were recorded on a Q-TOF Micro LC-MS mass spectrometer. All solvents were of analytical grade (Shanghai Chemical Plant, Shanghai, China). Commercial silica gel (Qing Dao Hai Yang Chemical Group Co., 200–300 and 400–600 mesh) was used for column chromatography, and precoated $SiO_2$ gel plates (Yan Tai Zi Fu Chemical Group Co., G60 F-254) were used for analytical TLC. Sephadex LH-20 gel (Amersham Biosciences) was also used for column chromatography.

**Plant Material.** The aerial parts of *C. cinerascens* were collected in Guangxi Zhuang Automomous Region, China, in July, 2008 and were identified by Jin-Gui Shen from Shanghai Institute of Material Medica. A voucher specimen is deposited at SIMM (voucher number JGL).

**Extraction and Isolation.** The air-dried, powdered aerial parts of *C. cinerascens* (2.5 kg) were extracted by exhaustive maceration in MeOH (3 × 5 L, for one week) at room temp. The extracts were concentrated under reduced pressure to afford 297.7 g of dried residues, respectively. The MeOH extract was partitioned successively between $CHCl_3$ and $H_2O$ to give $CHCl_3$ extract (30.1 g). The $CHCl_3$ residue was fractionated by gradient silica gel column chromatography (CC) eluting with a step gradient (0–100% EtOAc in light petroleum ether) to yield twelve fractions A–L. Fraction I was chromatographed by gradient Si gel CC (light petroleum ether/$CHCl_3$, from 9:1 to 3:7, followed by $CHCl_3$/MeOH, from 9:1 to 8:2) and further purify by Sephadex LH20 (light petroleum ether/$CHCl_3$/MeOH, 2:1:1) to give compound **1** (3.2 mg) and **2** (8.0 mg).

**Cipatrijugin G (1):** white powder; $[\alpha]_D^{25}$ + 4 (*c* 0.3, $CHCl_3$); IR (KBr) $\nu_{max}$ 3442, 2926, 2852, 1742, 1686, 1439, 1379 1250, 1032 $cm^{-1}$; $^1H$ and $^{13}C$ NMR data, see Table 1; ESIMS *m/z* 641.2 $[M + Na]^+$; positive ion HRESIMS: *m/z* 641.2205 (calcd for $C_{31}H_{38}O_{13}Na$, 641.2210).

**Cytotoxicity Assay.** Compounds **1** and **2** were evaluated for their cytotoxicity against human tumor cell line A549 by the MTT[21] method as previously reported.

## Electronic Supplementary Material

Supplementary material is available in the online version of this article at http://dx.doi.org/10.1007/s13659-013-0074-z and is accessible for authorized users.

## Acknowledgments

This research work was financially supported by the National Marine '863' Projects (No. 2013AA092902), the Natural Science Foundation of China (Nos. 21021063, 81273430, and 2107224), and was partially funded by the EU 7th Framework Programme-IRSES Project (No. 246987).

**Open Access** This article is distributed under the terms of the Creative Commons Attribution License which permits any use, distribution, and reproduction in any medium, provided the original author(s) and source are credited.

## Electronic supplementary material

Supplementary material, approximately 475 KB.
