# Supplementary material for: Cipatrijugin G, a new trijugin-type limonoid bearing an uncommon γ-hydroxybutenolide unit from the aerial parts of Cipadessa cinerascens
Source: Nat Prod Bioprospect. 2013 Nov 6;3(6):267–70. doi: 10.1007/s13659-013-0074-z (PMC4131602; doi:10.1007/s13659-013-0074-z)

Electronic Supplementary Material

Cipatrijugin G, a new trijugin-type limonoid bearing an uncommon γ-hydroxybutenolide unit from the aerial parts of *Cipadessa cinerascens*

Cheng-Shi JIANG,[a] Yan LI,[a] Zhen-Zhong WANG,[b] Xiao-Yin HUANG,[a] Wei XIAO,[b,*] and Yue-Wei GUO[a,*]

[a]State Key Laboratory of Drug Research, Shanghai Institute of Materia Medica, Chinese Academy of Sciences, Shanghai 201203, China

[b]Jiangsu Kanion Pharmaceutical Co. Ltd., Lianyungang 222001, China

Received 23 September 2013; Accepted 22 October 2013

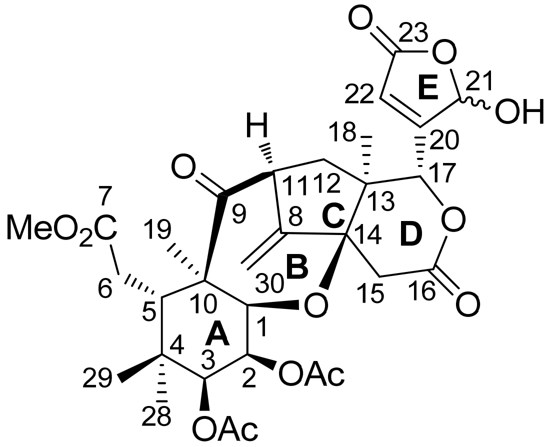

1

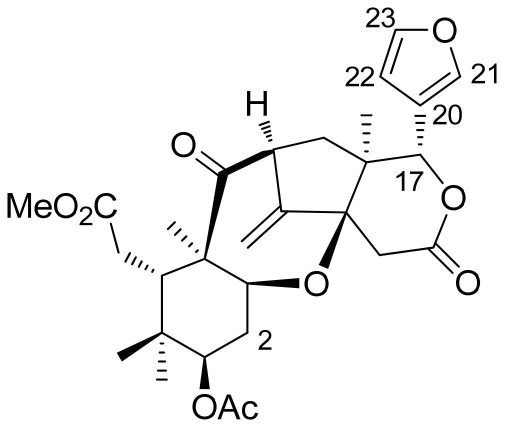

2

Structures of compounds **1** and **2**

*To whom correspondence should be addressed. E-mail: wzhzh-nj@163.net (W. Xiao); ywguo@mail.shcnc.ac.cn (Y.W. Guo)

S1. ^{1}H NMR spectrum of **1** ($CDCl_3$, 300 MHz)

S2. ^{13}C NMR spectrum of **1** ($CDCl_3$, 100 MHz)

S3. HSQC spectrum of **1**

S4. HMBC spectrum of **1**

S5. ^{1}H-^{1}H COSY spectrum of **1**

S6. ROESY spectrum of **1**

S7. ESI MS spectrum of **1**

S8. HRESI MS spectrum of **1**

S9. IR (KBr disk) spectrum of **1**

S1. ^{1}H NMR spectrum of **1** ($CDCl_3$, 300 MHz)

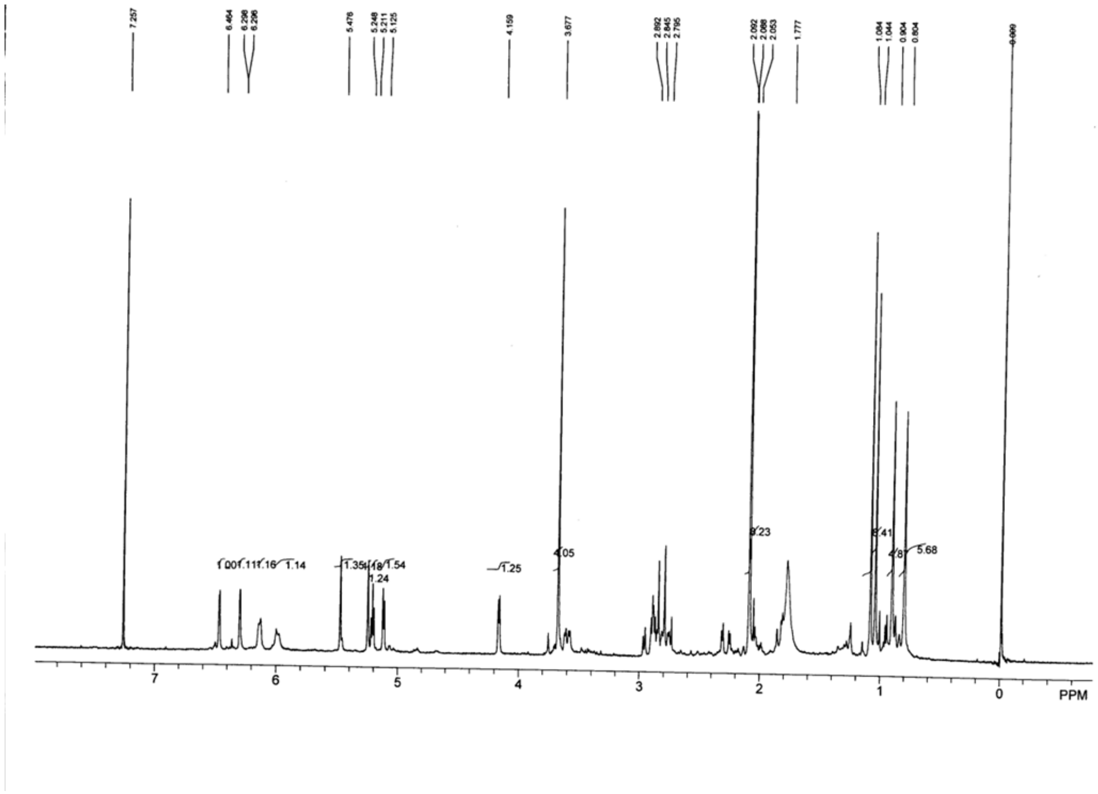

S2. ^{13}C NMR spectrum of **1** ($CDCl_3$, 100 MHz)

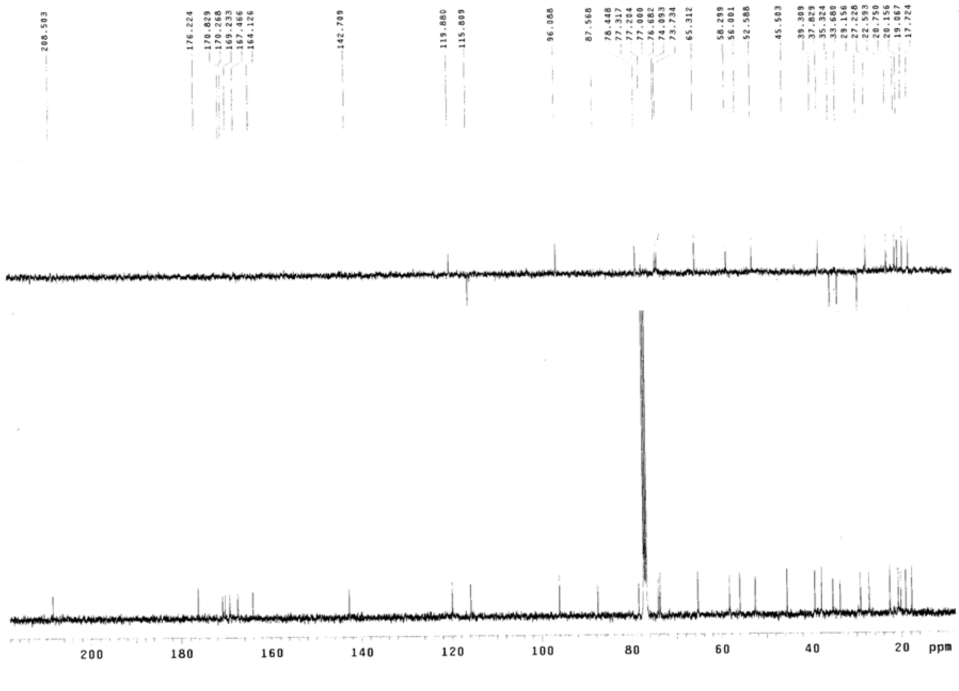

S3. HSQC spectrum of **1**

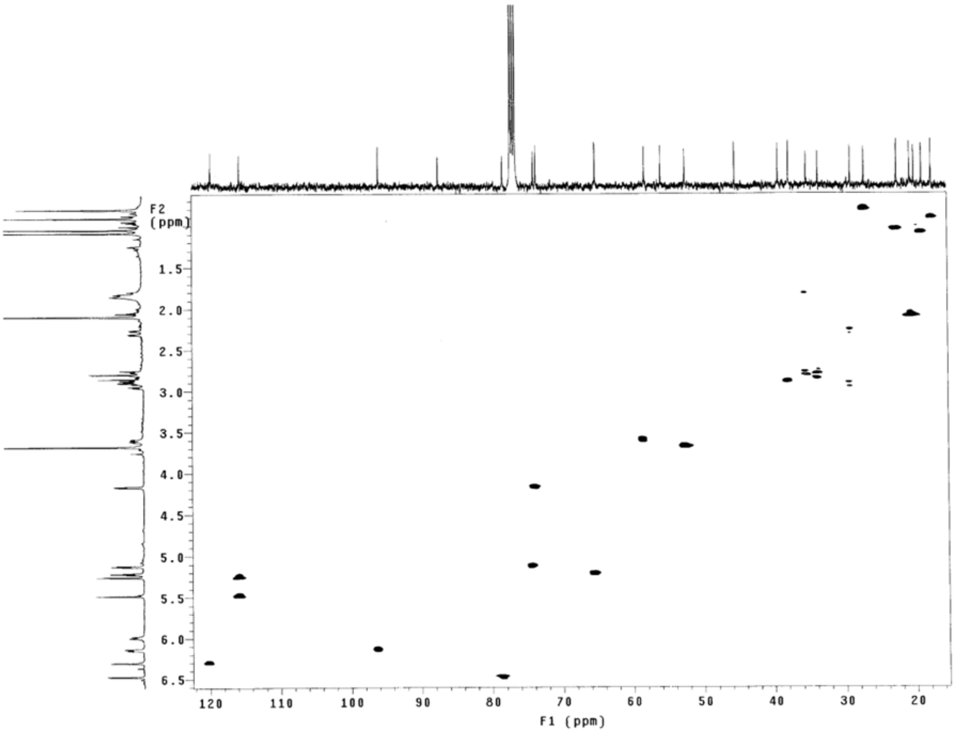

S4. HMBC spectrum of **1**

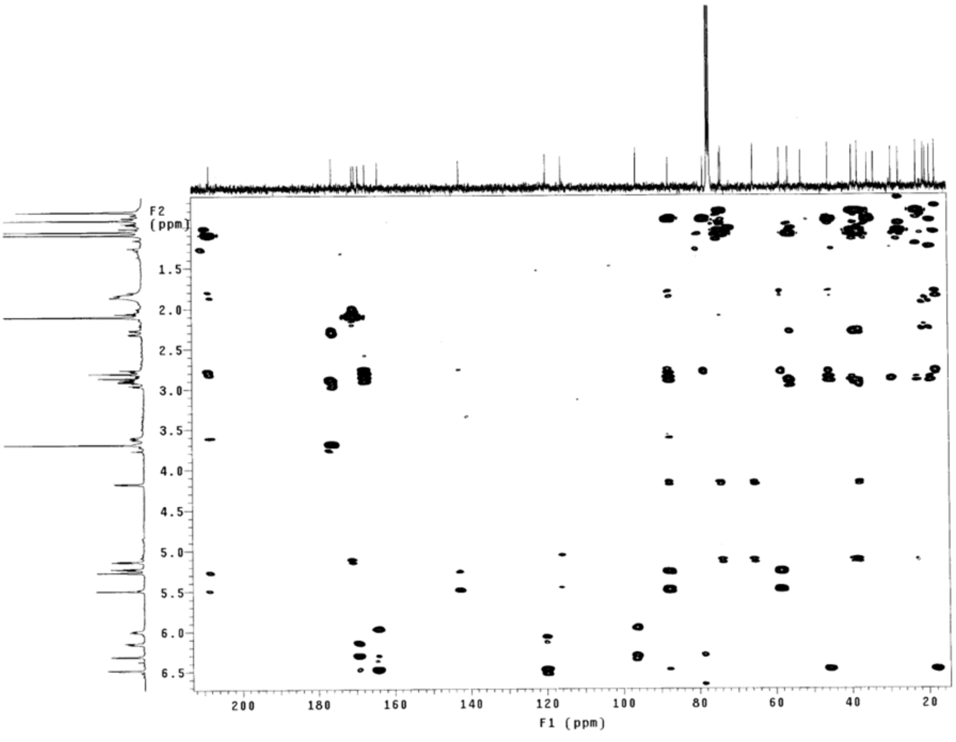

S5. ^{1}H-^{1}H COSY spectrum of **1**

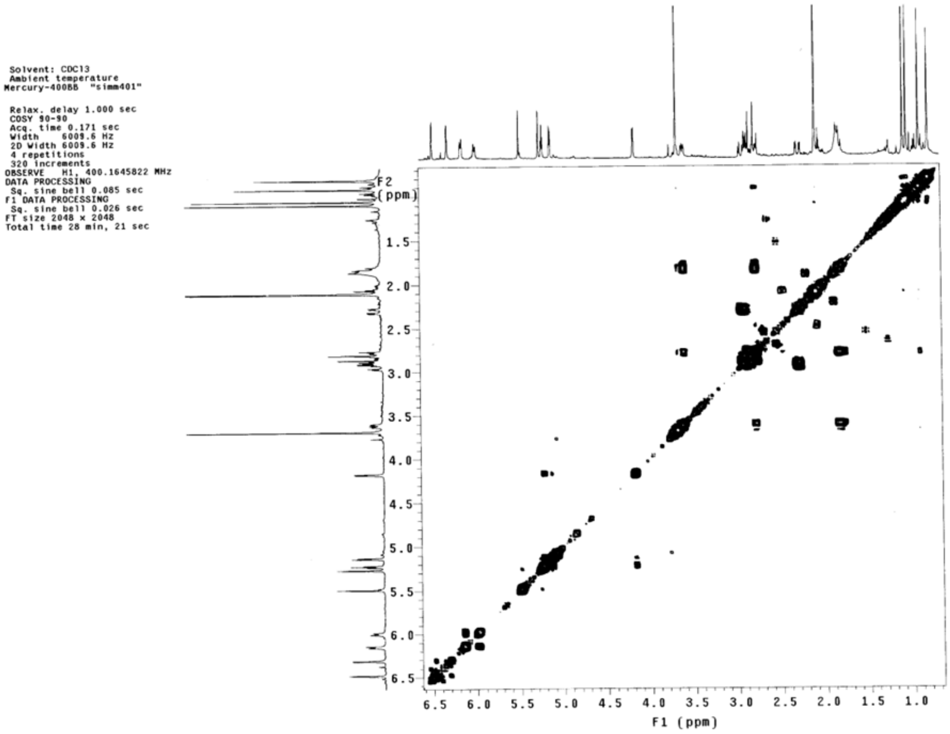

S6. ROESY spectrum of **1**

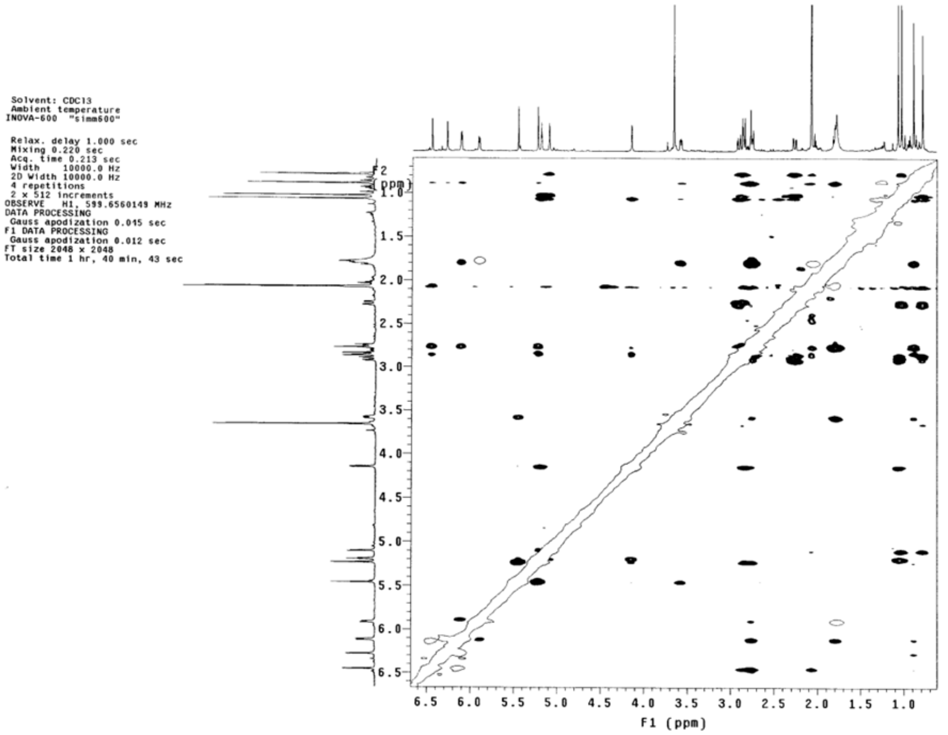

S7. ESI MS spectrum of **1**

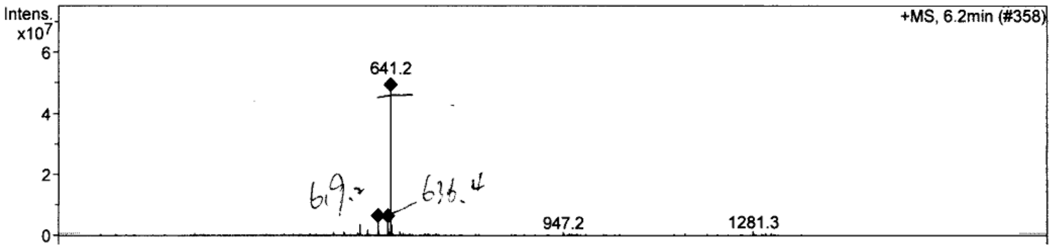

S8. HRESI MS spectrum of **1**

Elemental Composition Report **Page 1**

Tolerance = 50.0 PPM / DBE: min = -1.5, max = 50.0
Isotope cluster parameters: Separation = 1.0 Abundance = 1.0%

Monoisotopic Mass, Odd and Even Electron Ions
26 formula(e) evaluated with 1 results within limits (up to 20 closest results for each mass)

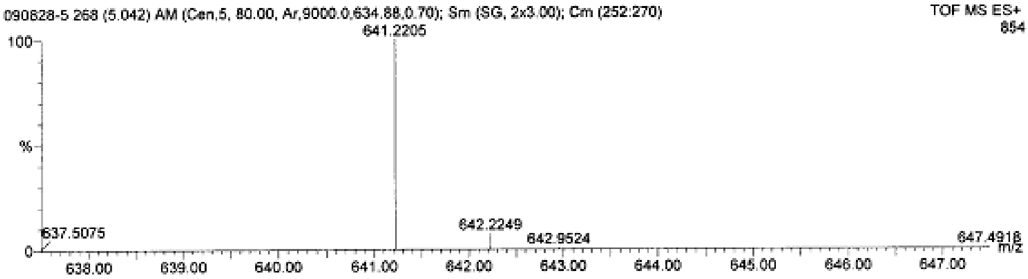

Minimum:	10.00				-1.5		
Maximum:	100.00		200.0	50.0	50.0		
Mass	RA	Calc. Mass	mDa	PPM	DBE	Score	Formula
641.2205	100.00	641.2210	-0.5	-0.8	12.5	1	C31 H38 O13 Na

S9. IR (KBr disk) spectrum of **1**

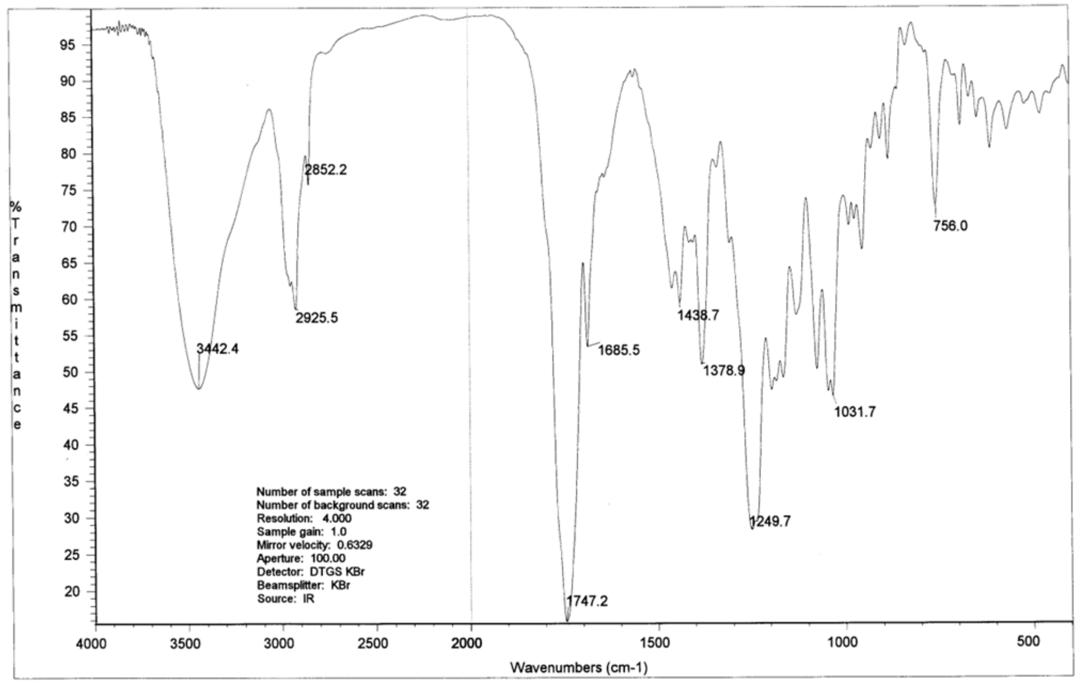

Supplement: Supplementary file 1 — Supplementary material, approximately 475 KB. [file 13659_2013_74_MOESM1_ESM.pdf]
